# The association between amalgam dental surfaces and urinary mercury levels in a sample of Albertans, a prevalence study

**DOI:** 10.1186/1745-6673-8-22

**Published:** 2013-08-29

**Authors:** Daniel J Dutton, Ken Fyie, Peter Faris, Ludovic Brunel, JC Herbert Emery

**Affiliations:** 1 Department of Community Health Sciences, University of Calgary, 3rd Floor, TRW Building, 3280 Hospital Drive NW, Calgary, AB T2N 4Z6, Canada; 2 Pure North S’Energy Foundation, Suite 800, 326 - 11th Ave. SW, Calgary, AB T2R-0C5, Canada; 3 Department of Economics, University of Calgary, Social Sciences Building, Room 554, 2500 University Dr. NW, Calgary, AB T2N 1N4, Canada

**Keywords:** Mercury, Urinary mercury, Dental amalgam, Canada

## Abstract

**Objective:**

The objective of this study was to quantify the relationship between number of dental amalgam surfaces and urinary mercury levels.

**Methods:**

This study uses participant data from a large philanthropic chronic disease prevention program in Calgary, Alberta, Canada. Urine samples were analysed for mercury levels (measured in μg/g-creatinine). T-tests were used to determine if differences in urine mercury were statistically significant between persons with no dental amalgam surfaces and one or more dental amalgam surfaces. Linear regression was used to estimate the change in urinary mercury per amalgam surface.

**Results:**

Urinary mercury levels were statistically significantly higher in participants with amalgam surfaces, with an average difference of 0.55 μg/g-creatinine. Per amalgam surface, we estimated an expected increase of 0.04 μg/g-creatinine. Measured urinary mercury levels were also statistically significantly higher in participants with dental amalgam surfaces following the oral administration of 2,3-dimercaptopropane-l-sulfonate (DMPS) and meso-2,3-dimercaptosuccinic acid (DMSA) which are used to mobilize mercury from the blood and tissues.

**Discussion:**

Our estimates indicate that an individual with seven or more dental amalgam surfaces has 30% to 50% higher urinary mercury levels than an individual without amalgams. This is consistent with past literature that has identified seven amalgam surfaces as an unsafe level of exposure to mercury vapor. Our analysis suggests that continued use of silver amalgam dental fillings for restorative dentistry is a non-negligible, unnecessary source of mercury exposure considering the availability of composite resin alternatives.

## Background

Amalgam dental surfaces are popular in dentistry and have been for decades. They contain approximately 50% mercury in combination with other metals including silver and copper [1]. The widespread use of amalgam surfaces can be attributed to their low cost and high durability compared to other types of surfaces. However, the use of dental amalgams has always been a source of controversy because of the potential health risks associated with exposure to mercury. The main concern over dental amalgams is the inhalation of mercury vapor from the surface, possibly leading to increased mercury levels in the body [1]. Richardson notes that viable alternative (non-Hg) restorative materials like composite resins exist, making reliance on dental amalgam for the restoration of carious teeth no longer necessary or essential [2]. This in turn means that continued use of dental amalgams for restorative dental care is a source of unnecessary exposure to mercury vapor which has been shown to exceed the safe level of mercury exposure when an individual has seven or more dental amalgam surfaces [2].

A sizeable body of research suggests that dental amalgams are a major source of mercury exposure in the general population, with several studies showing a correlation between the number of amalgam surfaces and brain, blood and urinary concentrations of mercury [3-13]. For example, it has been estimated that for every 10 amalgam surfaces an individual has, there is a corresponding increase in urinary mercury concentrations of approximately one μg/L, roughly doubling normal background concentrations [11]. Dental amalgams have also been correlated with disease outcomes, for instance, kidney function in children [14] or neurological degeneration in adults (e.g., Alzheimer’s disease) [15]. High levels of mercury in the diet, an allergy to mercury, impaired kidney function, and environmental or workplace exposure to mercury are all contraindications for dental amalgams according to Health Canada [16,17].

However, evidence to date is mixed, with some studies asserting that mercury levels are not elevated in those with dental amalgams, or that any observed elevations were not associated with health impacts [18-20]. The accumulated evidence on amalgam surfaces and the relationship with mercury levels is based on many small studies and few large studies, so it is possible that the wide-ranging conclusions in the literature are partly due to small sample sizes.

Recent Canadian estimates from a large, population-based sample show that, for Canadians with dental amalgams, over 80% are exposed to more mercury daily than the current government guidelines recommend [2]. This estimate was based on extrapolation from the number of observed amalgam surfaces per individual, and states that seven or more amalgams in an adult will lead to higher than recommended mercury levels per unit of body weight.

The objective of this study is to estimate the relationship between the number of amalgam surfaces and urinary mercury levels. We take advantage of a large sample of men and women with two measures for urinary excretion of mercury and number of amalgam surfaces. We find that individuals with one or more dental amalgam surfaces have, on average, at least double the urinary mercury level of individuals with no dental amalgam surfaces. We find a statistically significant linear relationship between urinary mercury levels and the number of amalgam surfaces. The magnitude of the exposure that we identify is consistent with past literature that has identified 7 amalgam surfaces as an unsafe level of exposure to mercury vapor [18]. Safety levels remain undefined for long-term, low-dose mercury exposure, but our analysis suggests that continued use of silver amalgam dental fillings results in higher mercury exposure that could be avoided with alternative filling materials.

## Methods

### Participants

Participants were drawn from enrollees in the Pure North S’Energy Foundation program (henceforth “Pure North”), which is a philanthropic wellness and chronic-disease prevention program based in Calgary, Alberta, Canada. Pure North assesses participant health status using questionnaires, biometric measurements and laboratory tests to provide personalized preventive health care services. Healthcare workers at Pure North (including nurses, doctors, and dentists) provide participants with lifestyle counselling, dietary supplementation with vitamins, minerals and other nutrients and dental care. There is no cost to the participants for the services offered - this includes dental care. As part of the health assessment, Pure North physicians assess the number of dental amalgam surfaces in an individual’s mouth and offer participants the option to test their urine mercury levels. We report data from 2,137 subjects from September 2010 through February 2013 who agreed to undergo a urinary six hour challenge to determine their mercury levels.

### Data collection

Dentists inspected participant’s mouths and oral x-rays to determine the number of amalgam fillings and surfaces. Typical molars have five surfaces, and consequently, there can be several amalgam surfaces per filled tooth (on average, there are 2 amalgam surfaces per filled tooth) [2]. Demographic and lifestyle information were obtained from health questionnaires routinely completed by Pure North participants. Height, weight, waist circumference and blood pressure were collected by the medical staff.

There are two measures of urinary mercury levels collected by Pure North. First, urine mercury levels are measured in a urine sample requiring no special considerations. Program participants are also offered the opportunity to undergo a 6 hour urinary challenge test for toxic metals (UTM challenge) which involves collecting urine for 6 hours following the oral administration of 30 mg 2,3-dimercaptopropane-l-sulfonate (DMPS) and 500 mg meso-2,3-dimercaptosuccinic acid (DMSA). DMPS and DMSA can be used to mobilize mercury from the blood and tissues and has been shown to significantly increase urinary excretion of mercury following a single administration [19]. While it remains unclear to what extent a single administration of DMSA and DMPS will mobilize stores of mercury in the brain, kidney and organs [19], it has been suggested that the use of this type of urinary challenge for the determination of urinary mercury levels increases the significance and reliability of urine levels as a measurement of mercury exposure and net bodily retention [18].

Post challenge with DMPS and DMSA, urine was collected in a 2.5-l polypropylene sampling vessel and a sample was collected from this vessel. The two collected urine samples were then sent to Doctor’s Data, Inc. (Champaign, IL) where they were stored at −20°C prior to testing. Analysis of urinary elements was performed by ICP-Mass Spectroscopy following acid digestion of the specimen. Urinary mercury concentrations are reported as μg/g-creatinine to reduce error introduced by variation in sample volume.

### Variables & statistical analysis

The primary outcome measures were urinary mercury levels measured from samples from the initial appointment and following the UTM challenge. Exposure to mercury was estimated based on the number of amalgam surfaces. Participant characteristics included age, sex, height, weight (expressed as tertiles), and BMI.

Analyses were completed using Stata 11. The variables used in this analysis were summarized with means and standard deviations if they were continuous variables and with percentages if they were binary or categorical variables. Comparisons of the average mercury levels by amalgam group were conducted with t-tests. We then used ordinary least squares regression models to investigate the association between mercury levels, amalgams, and patient characteristics. The initial model included age, sex, height in metres, weight in kilograms (expressed as tertiles), and BMI (weight/height^2^). For all tests of significance, we used a significance level of five percent to determine evidence of an association. Ethics approval for this study was obtained from the University of Calgary (Ethics ID E-24890).

## Results

**Figure 1:**
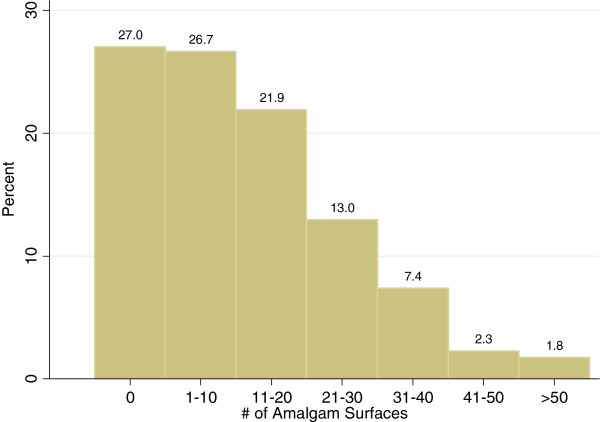
Frequencies of the number of amalgam surfaces in study participants.

Characteristics of the participants are reported in Table 1. The distribution of the number of surfaces per participant is presented in Figure 1. For participants with amalgams, the mean number of surfaces was 12.8 (median = 10). Comparing those with and without dental amalgams, we find statistically significant mean differences in both urinary mercury measures. Urinary mercury levels in participants with amalgams are 0.55 μg/g-creatinine higher, or 64% greater, than the mean of 0.86 μg/g-creatinine for participants without amalgams. UTM challenge urinary mercury levels for participants with one or more dental amalgam surfaces are on average five μg/g-creatinine higher – almost double – the mean of 5.32 μg/g-creatinine for those participants with no amalgam surfaces.

**Table 1:** Characteristics of study participants

**Characteristic**	**Summary measure (N = 2137)**	**Women (N=994) 47%**	**Men (N=1143) 53%**
Age in Years: Mean (sd)	49.3 (11.6)	49.7 (11.9)	48.9 (11.3)
1 or More Amalgams	73%	80%	67%
Mean Number of Amalgam Surfaces, inclusive of zero surfaces (sd)	12.8 (13.6)	14.4 (13.7)	11.4 (13.3)
Weight in kg: Mean (sd)	80.5 (17.4)	73.6 (16.8)	86.5 (15.6)
Weight <74kg	40%	61%	22%
Weight 74-90kg	33%	22%	41%
Urinary Hg (μg/g-creatinine): Mean (sd)	1.3 (1.8)	1.7 (2.3)	1.0 (1.2)
Urinary Challenge Hg (μg/g-creatinine): Mean (sd)	9.6 (12.9)	12.9 (15.1)	6.8 (9.9)

Notes: *sd* standard deviation.

**Figure 2:**
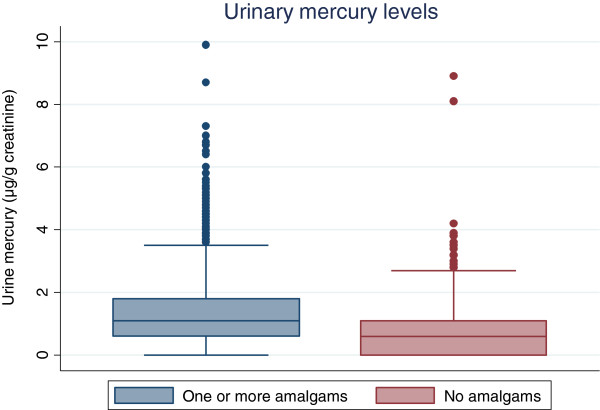
Frequencies of urine mercury levels in those with and without amalgams.

**Figure 3:**
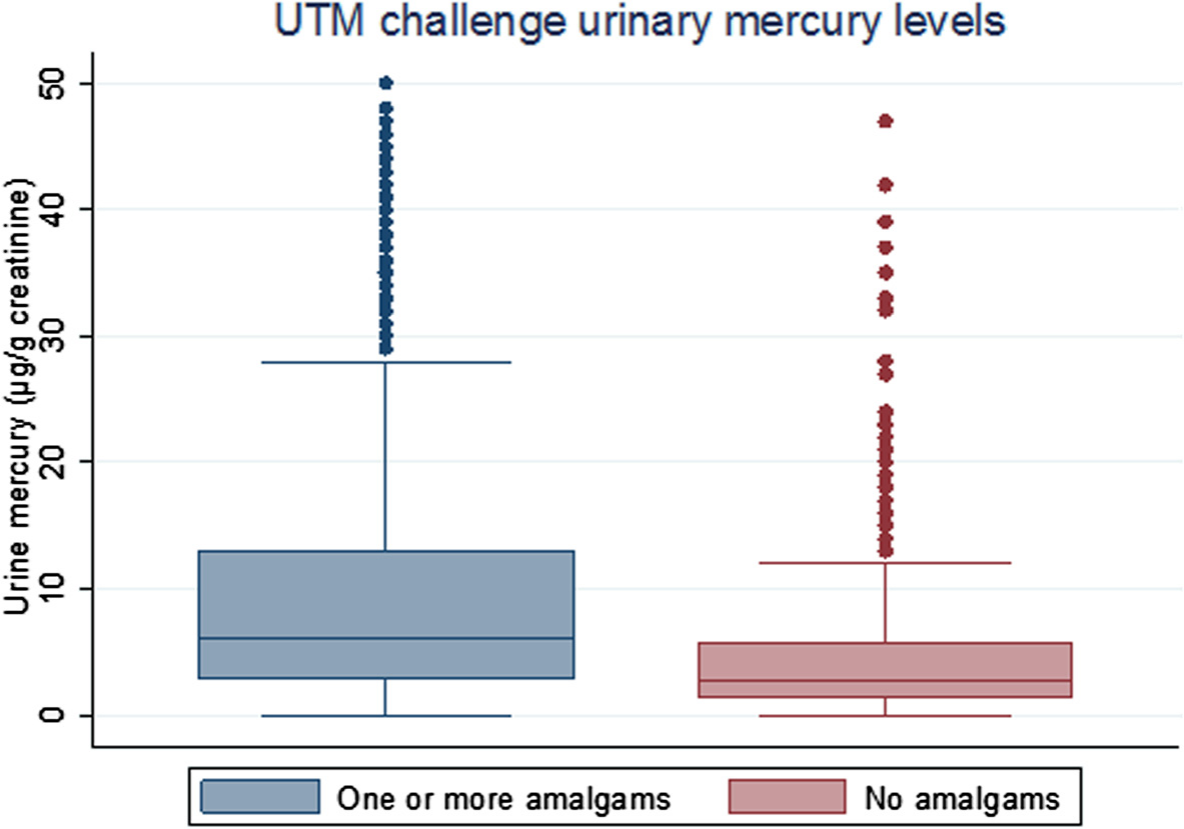
Frequencies of urine challenge mercury levels in those with and without amalgams.

Figure 2 shows the distribution of mercury levels for those with and without mercury amalgams and Figure 3 shows the same comparison using the UTM challenge urinary mercury measure. In both figures the median levels of μg/g-creatinine are nearly double for those with one or more amalgams than participants with no amalgam surfaces. The median mercury level for those with one or more dental amalgam surfaces was 1.1 μg/g-creatinine, versus 0.6 μg/g-creatinine for those with no amalgams. Meanwhile, the median UTM challenge urinary mercury level for those with amalgams was 6.3 μg/g-creatinine versus 2.75 μg/g-creatinine for those with no amalgam surfaces. Individuals with amalgams exhibited, in some cases, very high levels of mercury: there are more outliers in the amalgam surfaces group than the group without amalgam surfaces.

The results for the model of mercury levels, number of amalgams, and participant demographics are presented in Table 2. There was statistical evidence that sex and weight were associated with baseline and UTM challenge urinary mercury levels. With these characteristics and exposures in the model, there was no evidence that height, BMI or age confounded the relationship between amalgam surfaces and urinary mercury levels.

**Table 2:** Factors associated with urine mercury levels

	**Unprovoked mercury (μg/g-creatinine)**			**Provoked mercury (μg/g-creatinine)**		
	**Effect**	**95% CI**	**P**	**Effect**	**95% CI**	**P**
Baseline Level	0. 470 μg/g	0.314, 0.627	<0.001	1.73 μg/g	0.657, 2.806	<0.001
Per Surface	0.04 μg/g	0.03, 0.04	<0.001	0.32 μg/g	0.28, 0.35	<0.001
< 74 kg*	0.30 μg/g	0.10, 0.49	0.002	3.41 μg/g	2.11, 4.73	<0.001
74 to 90 kg*	0.15 μg/g	−0.04, 0.34	0.118	1.69 μg/g	0.40, 2.97	0.015
Female vs Male	0.52 μg/g	0.31, 0.63	<0.001	4.14 μg/g	3.04, 5.24	<0.001

* baseline group: >90 kg. Other than the baseline level, the effect indicates the unit increase in mercury level associated with each participant characteristic.

The coefficient estimate for the per-amalgam surface increase in urinary mercury of 0.04 ug/g-creatinine is statistically significant. For males and females of mean weight in the sample, this per surface increase represents a 5% and 3% increase in ug/g-creatinine over the baseline level for someone with no amalgam surfaces. For the UTM challenge urinary mercury measures, the per surface effect of 0.32 ug/g-creatinine represents 9% and 4% increases over the ug/g-creatinine for someone with no amalgam surfaces.

## Discussion

We find a statistically significant association between amalgam surfaces and two measures of urinary mercury levels. Other researchers have examined the relationship between amalgam surfaces and urinary concentrations [4,6-10,13] but only two [18,20] have specifically examined UTM challenge urine mercury levels, with mixed results. Aposhian et al. measured mercury excretion nine hours after administration of a single dose of 300 mg DMSA in 14 patients with or without dental amalgams [18]. They found that individuals with amalgams excreted roughly 3 times more mercury after DMSA than those without amalgams and that there was a statistically significant positive relationship between mercury excretion and total amalgam surface area. In contrast, Vamnes et al. found no significant relationship between number of amalgam surfaces and urinary mercury excretion following DMPS injection in 41 patients, although they note that subjects in the study were relatively similar in terms of number of amalgams [20]. In both cases the conclusions that could be drawn from these studies were limited by their small sample sizes. A main strength of our study is that the Pure North program gathered data on over 2000 individual participants. This much larger sample size enabled us to determine the relationship between amalgam surfaces and urinary concentration while considering other confounders mentioned in the literature.

Exposure to mercury vapor is a known health risk with no clearly established safe level of exposure. Health Canada has previously acknowledged that dental amalgam is the single largest source of mercury exposure for the average Canadian but assesses that other than for the two to three percent of the population with mercury allergies or hypersensitivity, evidence does not indicate that dental amalgam exposures are high enough to cause illness [21]. Compared to the urinary mercury level associated with clinical mercury poisoning of 100 ug/g-creatinine, our estimates of elevated urinary mercury due to amalgam surfaces are well below that level and would be considered “not harmful” by Health Canada.

Chronic exposure to low doses of mercury from dental amalgam is difficult to evaluate from a health safety perspective and there is no established level of mercury exposure considered safe or unsafe over longer periods of time. The Tolerable Daily Intake (TDI) concept for exposure to hazardous substances defines a level of exposure “to which people could be exposed continuously over their lifetime without suffering any harmful effects”. TDI’s for mercury are based on exposures from industrial and environmental sources [21]. Mercury vapor doses from dental amalgam fillings by industrial standards are considered to be small at any given point in time, but given that exposure to amalgams is essentially lifelong it can be argued that the current thresholds for mercury exposure should be lowered [22]. Recent research on rats has speculated on particular mechanisms through which low, constant exposure to mercury negatively affected body systems [23,24]. A review on the health of humans and low levels of mercury concluded that the evidence base was too small to quantify the risk of chronic exposure to mercury [25].

According to Richardson, individuals with seven amalgam surfaces or more would be experiencing levels of urinary mercury that correspond to exposures above the federal government’s recommendation for safe levels of mercury vapor [2]. Our estimates of the per-amalgam surface increase in μg/g-creatinine are consistent with the ranges estimated by Richardson for the Canadian population (if not slightly higher) using the Canadian Health Measures Survey, thus, the mercury exposure levels associated with amalgams estimated by Richardson can be used with our data [2]. Our model shows that seven amalgam surfaces would be expected to increase urinary mercury levels by 36% over that of an average male with no amalgam surfaces and by 22% over that of an average weight female with no amalgam surfaces. For the UTM challenge urinary mercury measure, compared to no amalgam surfaces, having seven surfaces would increase the urinary mercury concentration by 65% in males of mean weight for the sample and 30% in females of mean weight for the sample. As the mean number of amalgam surfaces observed in our dataset was 12.8, the majority of persons in our sample are experiencing exposure to mercury vapor that Richardson has shown to be above Health Canada’s recommended safe level [2].

Given this clear association between amalgam surfaces and urinary mercury levels, it is important for future research to address the impact that mercury vapor exposure from amalgam surfaces may have on health outcomes. The continued widespread use of amalgams could be causing negative health consequences in the Canadian population. Dental amalgam has a unique regulatory treatment. Health Canada describes dental filling materials as being classified as medical devices under the Medical Devices Regulations of the Food and Drugs Act and the authority of that act applies to sale of the device but not its use by dentists. Where medical devices to be implanted in the body must pass a pre-market review of safety and efficacy data, dental materials including amalgam are exempted from this review since they had been on the market before the enactment of the safety regulation. Consequently, dental amalgam remains in use until evidence of harm to health is produced as opposed to the usual practice of not being in use until evidence of safety is produced [21].

Health Canada’s 1996 Position Statement on Dental Amalgam assessed that there was not sufficient evidence to support a total ban on amalgam or the removal of sound amalgam fillings in patients who have no indication of adverse health effects attributable to mercury exposure. Even in the absence of clinical evidence of adverse health effects, Health Canada did recommend reducing exposure to heavy metals provided the reductions can be achieved “at reasonable cost and without introducing other adverse effects”. Health Canada recommended the avoidance of amalgam fillings for primary teeth of children, for pregnant women and people with kidney disease. Health Canada also recommended that dentists provide patients with sufficient information on the risks and benefits of dental materials for filling teeth so that patients can make informed choices about what to have implanted in their mouths. Health Canada also expressed that dentists should acknowledge the patient’s right to decline treatment with any dental material.

There was a good deal of unexplained variation in our model. Even with the inclusion of additional patient characteristics and exposures, the model was only able to account for approximately 10% to 22% of the variation in the baseline urinary and post challenge urinary mercury levels. This variation could be due to a number of factors, including unmeasured exposures, measurement error, how many years the amalgam has been in the bearer’s mouth, the condition of the amalgam from wear and tear and metabolic factors. Research has shown that there are many genetic polymorphisms in the genes for enzymes and proteins involved in the detoxification and elimination of heavy metals, and that this can influence mercury levels. Variations in the genes for glutathione related enzymes and selenoproteins have both been shown to significantly influence inter-individual variations in mercury retention, elimination and overall mercury burden [26]. There is also evidence that gum chewing (especially nicotine gum) can increase mercury levels in persons with mercury amalgams. In addition, there may be numerous possible environmental sources, including fluorescent light bulbs, consumption of large fish, and electronics. The fact that the number of dental amalgam surfaces has a sizeable statistically significant correlation with urinary mercury levels across many individuals means that even without explaining total variation in mercury levels we can identify an important source of mercury exposure.

One limitation of our study is that, in general, it is difficult to interpret the meaning of post challenge urine levels. The UTM challenge used to obtain the second urine measure analysed in this study will draw mercury from blood and other tissues. To our knowledge, only one study has examined the relationship between UTM challenge urinary mercury concentrations and blood mercury levels [21]. In addition, the impact of metal binding agents used for the UTM challenge may vary from person to person and participants may have made errors in the urine collection. Furthermore, differences in urine flow rate, which is affected by fluid intake and time of day, could impact mercury concentration in the urine. Trachtenberg et al. found that increased urine flow rate was associated with a significant decrease in mercury concentration and a non-significant increase in mercury excretion rate [27]. However, the mercury/creatinine ratio was unaffected by flow rate, suggesting that using creatinine-corrected mercury concentrations could compensate for differences in flow-rate or fluid intake. Furthermore, all measures of mercury significantly increased with the number of amalgam surfaces, regardless of variation due to flow-rate. Thus, despite these drawbacks, our large sample size provides convincing evidence of a statistically important association between dental amalgam surfaces and urinary mercury levels.

Another limitation to our study is that we are unable to measure the age of the amalgam surfaces in participants. It has been shown that as an amalgam surface ages the amount of mercury contained in the surface decreases due to chemical change [28]. While the average age of participants in our study is approximately 49 years, it is possible that some of the amalgam surfaces observed were new and thus gave off more mercury than the average amalgam surface. More likely, considering the average age of participants, is that the amalgam surfaces we observed are older and thus the mercury levels we observed are below the level of mercury vapor exposure associated with the average aged amalgam surface. This means that our estimate of an unsafe number of amalgams, which we derived from a previously published study [2], may apply only to older and less potent amalgam surfaces.

Our study does not discuss the effect on mercury urine of amalgam removal. Individuals with zero amalgam surfaces have lower urine mercury levels than individuals with one or more amalgam surfaces. However, that does not imply that removing amalgams from the amalgam group will result in urine mercury levels similar to individuals without amalgams. Some studies have shown that removing amalgams is associated with a temporary spike in mercury levels at the time of removal, after which mercury levels tend to decrease to levels below the pre-removal mercury level; that spike that is attributed to agitation of the mercury in the dental amalgam [29,30].

## Competing interests

Fyie and Brunel are employed by the service provider that supplied the raw data.

## Authors’ contributions

DJD, JCHE, KF and LB were responsible for writing and editing the manuscript. DJD, KF, JCHE and PF were responsible for the data management and statistical analysis. LB clarified testing protocols used by Pure North. All authors read and approved the final manuscript.
